# Inhibition of Ovarian Epithelial Carcinoma Tumorigenesis and Progression by microRNA 106b Mediated through the RhoC Pathway

**DOI:** 10.1371/journal.pone.0125714

**Published:** 2015-05-01

**Authors:** Shuo Chen, Xi Chen, Yin-Ling Xiu, Kai-Xuan Sun, Yang Zhao

**Affiliations:** Department of Gynecology, the First Affiliated Hospital of China Medical University, Shenyang, 110001, P.R. China; The University of Hong Kong, CHINA

## Abstract

Epithelial ovarian cancer (EOC) is the most lethal of the gynecological malignancies. Exploring the molecular mechanisms and major factors of invasion and metastasis could have great significance for the treatment and prognosis of EOC. Studies have demonstrated that microRNA 106b (miR-106b) may be a promising therapeutic target for inhibiting breast cancer bone metastasis, but the role of miR-106b in EOC is largely unknown. In this work, miRNA-106b expression was quantified in various ovarian tissues and tumors. Ovarian carcinoma cell lines were transfected with miR-106b, after which, cell phenotype and expression of relevant molecules was assayed. Dual-luciferase reporter assays and xenograft mouse models were also used to investigate miR-106b and its target gene. MiR-106b mRNA expression was found to be significantly higher in normal ovarian tissues and benign tumors than in ovarian carcinomas and borderline tumors (*p *< 0.01), and was negatively associated with differentiation (Well *vs*. Por & Mod) and the International Federation of Gynecology and Obstetrics (FIGO) staging (stage I/II *vs*. stage III/IV) in ovarian carcinoma (*p *< 0.05). MiR-106b transfection reduced cell proliferation; promoted G1 or S arrest and apoptosis (*p *< 0.05); suppressed cell migration and invasion (*p *< 0.05); reduced Ras homolog gene family member C (RhoC), P70 ribosomal S6 kinase (P70S6K), Bcl-xL, Matrix metallopeptidase 2 (MMP2), MMP9 mRNA and protein expression; and induced p53 expression (*p *< 0.05). Dual-luciferase reporter assays indicated that miR-106b directly targets RhoC by binding its 3’UTR. MiR-106b transfection also suppressed tumor development and RhoC expression *in vivo* in xenograft mouse models. This is the first demonstration that miR-106b may inhibit tumorigenesis and progression of EOC by targeting RhoC. The involvement of miR-106b-mediated RhoC downregulation in EOC aggression may give extended insights into molecular mechanisms underlying cancer aggression. Approaches aimed at overexpressing miR-106b may serve as promising therapeutic strategies for treating EOC patients.

## Introduction

Ovarian cancer is the most lethal of the gynecological malignancies, but relatively little is known about the molecular genetics of its initiation and progression. The primary genetic alterations associated with epithelial ovarian cancer, which accounts for 90% of ovarian cancer, remain to be identified [1]. Recurrence and metastasis seriously affect the prognosis of ovarian cancer; the 5 year survival rate for all stages of the disease has been estimated to be 35–38%. Due to this high mortality rate, exploring the molecular mechanisms of ovarian epithelial carcinoma initiation and development, and identifying the major factors of invasion and metastasis, could have great significance for treatment and prognosis.

MicroRNAs are non-coding single-stranded RNAs of ~22 nucleotides in length that constitute a novel class of gene regulators. They negatively regulate gene expression at the post-transcriptional level by binding to imperfect complementary sites in the 3’-UTR (untranslated region) of their target messenger RNA transcripts [2]. The binding of miRNAs to target mRNAs leads to translational repression, or decreases the stability of the mRNA. MiRNAs control various biological processes including cell differentiation, cell proliferation, apoptosis, drug resistance and fat metabolism [3–4]. MiR-106b is transcribed from the miR-106b-25 cluster located on chromosome 7. It has been reported that miR-106b-25 cluster might play a vital role in the pathogenesis of renal fibrosis. They found that expression of miR-106b during TGF-β1-induced EMT was inhibited by Sal B (Salvianolic acid B) in vitro; miR-106b attenuates EMT in renal fibrosis via inhibitory effects on TGF-β type II receptor expression and the TGF-β signaling pathway [5]. Khuu C et al. Found that high levels of miRNAs encoded by the miR-106a-363 cluster may contribute to inhibition of proliferation by decreasing expression of several sibling miRNAs encoded by miR-17-92 or by the miR-106b-25 cluster [6]. Sampath D et al. showed that low expression of miR106b may offer chronic lymphocytic leukemia (CLL) cells a mechanism whereby the apoptotic potential of p73 is repressed. Chemotherapeutic drugs that activate miR106b could potentially circumvent the resistance associated with p53 dysfunction in CLL [7]. Ni XJ et al. found that MiR-106b levels in orthotopic tumor tissue showed a negative correlation with MMP2 (matrix metalloproteinase 2) expression and breast cancer bone metastasis [8]. Thus, miR-106b may play vital role in many cancers through diverse mechanisms. However, the targets of miR-106b in epithelial ovarian cancer have not yet been determined and were therefore investigated in this study.

## Materials and Methods

### Cell line culture and transfection procedure

Ovarian cancer cell lines OVCAR3, HO8910-PM (highly invasive HO8910) were obtained from the American Type Culture Collection (ATCC), SKOV3/DDP (cisplatin-resistant SKOV3) was purchased from Tumor Cell Bank of the Chinese Academy of Medical Science (Peking, China), and they were all serous cystic adenocarcinoma cells. Cells were cultured in RPMI-1640 medium containing 10% fetal bovine serum (FBS) and 1% antibiotic-antimycotic solution (100 U/mL penicillin and 100 μg/mL streptomycin). Cells were maintained at 37°C in a humidified atmosphere containing 5% CO_2_. MiR-106b mimics or non-targeting control mimic (Mock) were transiently transfected into the three ovarian cell lines using Lipofectamine 2000, following the manufacturer’s protocol. The miR-106b-5P homo target sequence was: 5'-UAA AGU GCU GAC AGU GCA GAU-3’.

### Cell proliferation assay

Cells were seeded in 96-well plates at 2.0×10^3^ cells per well, transfected with miR-106b or mock mimic and further incubated for 0, 24, 48 or 72 h. Cell proliferation was evaluated using Cell Counting Kit-8 (CCK-8) (Dojindo, Tokyo, Japan) according to the manufacturer's instructions. 10 μL CCK-8 was added to each well for 4 h, after which optical density values (OD) were measured at 450 nm.

### Cell cycle analysis

Cell cycle analysis was performed after 72h incubation. Cells were collected, washed with PBS and fixed with 75% ice-cold ethanol, then treated with 250μg/mL RNase at 37°C for 30 min. Propidium iodide (PI, KeyGen, Nanjing, China) was added to a final concentration of 50μg/mL for 30 minutes in the dark. Immdiatelly, flow cytometry was used to examine PI signal.

### Flow cytometric apoptosis assay

Flow cytometry was performed to detect phosphatidylserine externalization as an endpoint indicator of early apoptosis, using cells stained with PI and FITC-labeled Annexin V (KeyGen, Nanjing, China) according to the manufacturer's protocol. Briefly, after incubation for 72 h, cells were washed with PBS twice and harvested, resuspended in 1×binding buffer at a concentration of 1×10^6^ cells/mL. All the cells were incubated with 200 μL binding buffer (containing 10 μL Annexin V-FITC) in the dark for 15min at room temperature and then 300μL binding buffer (containing 5 μL PI) were added to each tube. Flow cytometry was performed within 1 h.

### Wound healing assay and cell invasion assay

Wound healing assay was performed to investigate cell migration ability in vitro. Briefly, cells transfected with or without miR-106b mimic were cultured in six-well plates; linear wounds were created using a 200 μL pipette tip. Cells were photographed and the wound area was measured by Image J software (National Institutes of Health, Bethesda, MD, USA) after 0, 12, 24, 48, and 72h incubation (n = 3). Wound healing rate was calculated as: [(the area of the original wound—the area of the actual wound at different times) / the area of original wound], expressed as a percentage.

In addition, transwell assay was performed to evaluate cell mobility. Transwell chambers of 6.5 mm (BD Bioscience, San Jose, CA, USA) were placed in 24-well culture plates which separated the plates into upper and lower chambers. 40 μL of 8 mg/mL Matrigel (Becton-Dickinson Labware, Bedford, MA, USA) was placed on the upper surface to simulate a matrix barrier for the invasion assay. 600 μL RPMI-1640 with 10% FBS was in the lower chamber while 200 μL RPMI-1640 containing 5×10^4^ cells was placed in the upper chamber. After incubation for 72 h, cells that had migrated through the membrane were fixed stained with 0.1% crystal violet for measurement.

### Subjects

Human clinical samples were collected from surgical specimens from the Department of Gynecology, The First Affiliated Hospital of China Medical University (Shenyang, Liaoning, China), between 2003 and 2011. Samples were including normal ovarian tissue, ovarian epithelial benign tumors (serous and mucinous cystadenocarcinoma), borderline tumors, primary carcinoma and metastatic omentum. All specimens were histopathologically diagnosed on the basis of the 2009 FIGO staging system. Samples were frozen immediately and preserved at -80°C. The samples were obtained from patients who never underwent chemotherapy, radiotherapy or adjuvant treatment before surgery. Each of the patients participating this study obtained written informed consent, and this study was approved by the China Medical University Ethics Committee.

### Real-time reverse transcriptase PCR

Total RNA of all the samples and cell lines were extracted using TRIzol (Takara, Shiga, Japan) following the manufacturer’s instructions. Using avian myeloblastosis virus transcriptase and random primers (Takara, Shiga, Japan), total RNA (2μg) was reversely transcribed. The cDNA obtained was performed to undergoing real-time PCR amplification in 20μL reactions using the SYBR Premix Ex Taq II kit (Takara, Shiga, Japan). The oligonucleotide primers for PCR came from GenBank sequences and the threshod (ddCt) values were calculated after normalization to GAPDH (glyceraldehyde-3-phosphate dehydrogenase).

### Western blotting

Equal amount of total proteins (60μg) was separated with sodium dodecyl sulfate-polyacrylamide gels, transferred to Hybond membranes (Amersham, Munich, Germany). The membranes were incubated for 1h with 5% skimmed milk in Tris-buffered saline with Tween 20 (TBST). Immunobloting was performed by incubating with antibodies against p53, P70S6K, RhoC, Bcl-xL, MMP2, or MMP9 (Santa Cruz Biotechnology, Santa Cruz, CA, USA) overnight. After rinsed with TBST, the membrane was incubated with anti-mouse, anti-rabbit, or anti-goat IgG antibodies (1:1000; Dako, Carpinteria, CA, USA) for 2h. Immunoreactive bands were detected with X-ray film (ImageQuant LAS 4000, Fujifilm, Tokyo, Japan) and ECL Plus detection reagents (Santa Cruz Biotechnology, Santa Cruz, CA, USA).

### Dual-luciferase reporter assay

Based on the human RhoC mRNA sequence in GeneBank, a pair of annealing oligonucleotides, which contained the putative binding site for miR-106b, were designed and synthesized by Sangon Biotech (Shanghai, China). The double-stranded annealing products were inserted downstream of the firefly luciferase reporter in the pmirGLO Dual-luciferase miRNA target expression vector (Promega, USA). A pair of mutant annealing oligonucleotides containing a mutant sequence in the seeding region of miR-106b was also cloned into the same region of the vector. HEK 293T cells were plated in 24-well plates for 24 h, then co-transfected with 50 nM of miRNA-106b mimic, or the mock miRNA, and 600 ng dual-luciferase vector, which contained either wild-type or mutant RhoC-3’-UTR. 48 h after transfection, luciferase activity was measured using the Dual-Luciferase Reporter Assay System (Promega). Both renilla luciferase activity and firefly luciferase activity were measured, and relative luciferase activity was normalized to the renilla luciferase activity. Each experiment was repeated at least three times.

### In vivo xenografts

Female BALB/C nude mice, aged 5 weeks, were purchased from Beijing HFK Bioscience Co., Ltd (Beijing, China) for tumorigenicity assays. The animals were randomly divided into two groups (five mice per group) and maintained in a specific pathogen-free environment with a daily 12 h light/12 h dark cycle, at 22±2°C. Skin tumor xenografts in the nude mice were established by subcutaneous injection of 200 μL PBS containing 5×10^6^ exponential-growth OVCAR3 cells, with or without miR-106b transfection. Tumor volume was measured every three days and calculated according to the formula: TV (mm3) = length × width2 × 0.5. At week 9 after tumor induction, all the mice were sacrificed by CO2 inhalation, since the maximum tumor volume of 1.0 cm3 was established to minimize other influence factors. All animal manipulations were performed following the National Institutes of Health Guide for the Care and Use of Laboratory Animals, and were approved by the China Medical University Animal Care and Use Committee (Shenyang, Liaoning, China). Our in vivo studies in accordance with the ARRIVE guidelines (S1 Table).

### Immunofluorescent staining

Immunofluorescent staining was performed following a standard protocol (Santa Cruz Biotechnology). Briefly, a 5 μm frozen section from each sample was fixed in acetone at 4°C overnight. After washing with PBS for three times, sections were blocked with 1% bovine serum albumin (BSA) for 30 min and incubated overnight at 4°C with mouse anti-human RhoC primary antibody (1:50). Following PBS washing, the sections were incubated with anti-mouse immunoglobulin G (IgG)–FITC (1:100, Santa Cruz Biotechnology, CA, USA) for 2 h at room temperature in the dark, then washed again with PBS. Nuclei were stained with DAPI (1 μg/mL, Sigma-Aldrich) for 30 min at room tempreture. Coverslips were mounted with SlowFade Gold Antifade Reagent (Invitrogen, Carlsbad, CA, USA) and all the sections were imaged using a laser confocal microscope (Olympus, Tokyo, Japan).

### Statistical analysis

SPSS 17.0 software (SPSS, Chicago, IL, USA) was employed and data was performed as means ± SEM and compared using the Mann—Whitney U test or paired samples t-test. P value < 0.05 indicated statistically significant.

## Results

### Correlation of miR-106b mRNA expression with pathogenesis and aggression of ovarian carcinoma

**Fig 1:**
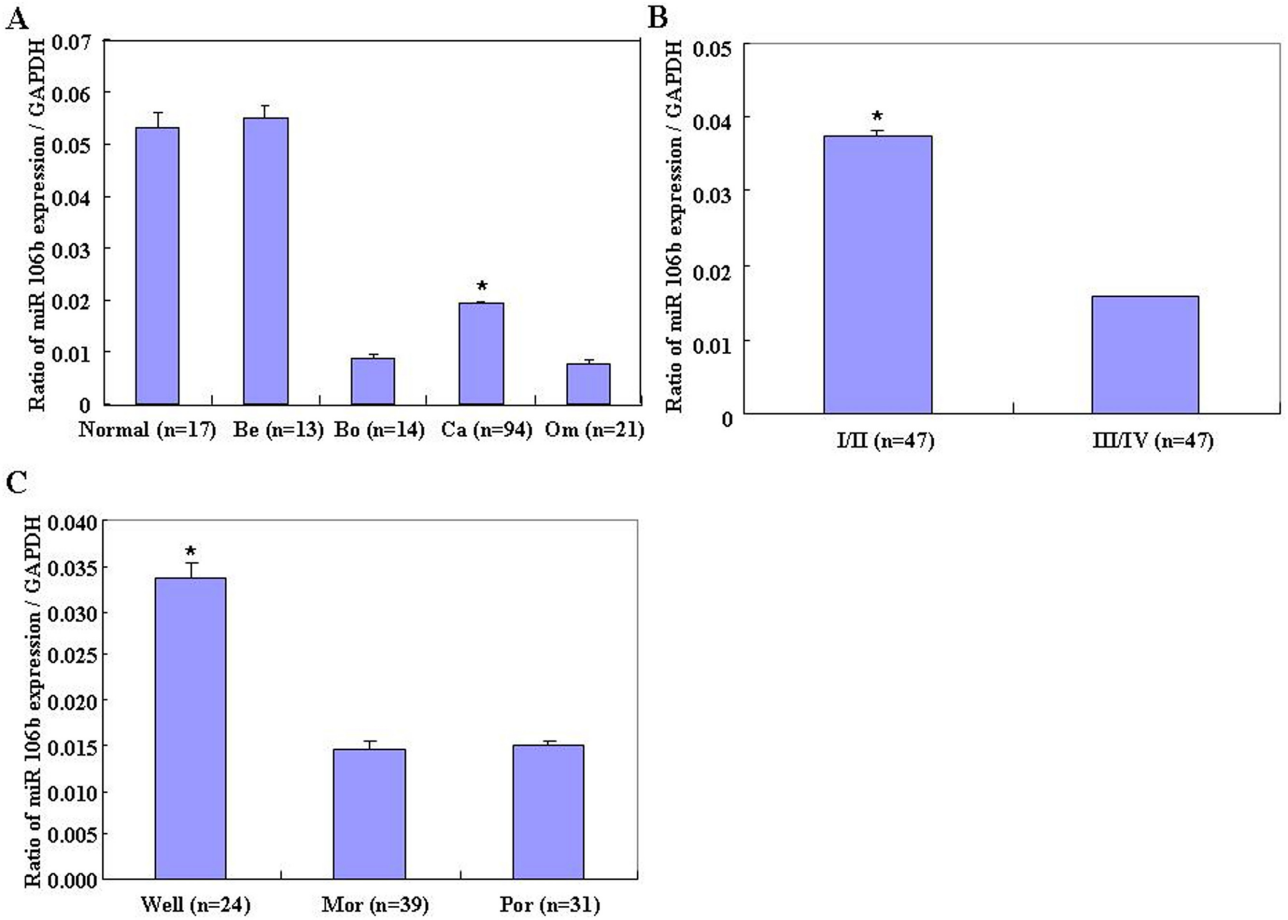
Correlation of miR-106b mRNA expression with ovarian carcinoma pathogenesis and aggression. (A) MiR-106b mRNA expression was significantly lower in ovarian carcinomas and borderline tumors than in normal ovarian tissues and benign tumors and lower in metastatic omentum than in primary ovarian carcinomas. MiR-106b mRNA expression was negatively associated in ovarian carcinoma with (B) International Federation of Gynecology and Obstetrics (FIGO) stage (stage I/II vs. stage III/IV), (C) differentiation (well vs. poor and moderate).

MiR-106b was quantified in normal ovarian tissue, benign and borderline tumors, and primary carcinoma using real-time PCR. Its expression was found to be significantly higher in normal ovarian tissues and benign tumors than in ovarian carcinomas and borderline tumors (Fig 1A, *p* < 0.01), and was negatively associated with FIGO stage (stage I/II *vs*. stage III/IV, Fig 1B, *p* < 0.05) and differentiation (Well *vs*. Por & Mod, Fig 1C, *p* < 0.01) in ovarian carcinoma.

### Effects of miR-106b transfection on phenotype in ovarian carcinoma cells *in vitro*

**Fig 2:**
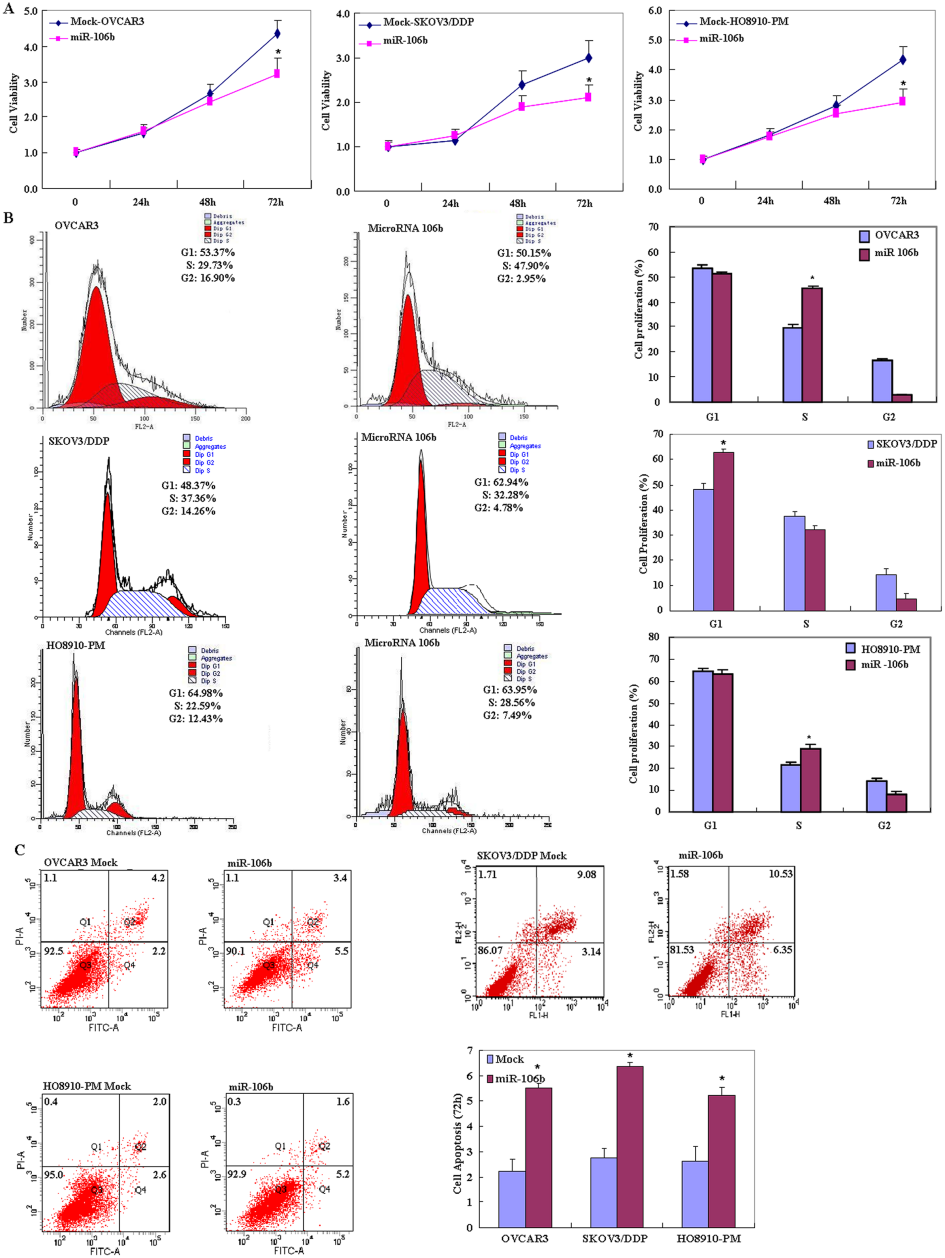
Effects of miR-106b transfection on ovarian carcinoma cell proliferation, cell cycle and apoptosis of ovarian carcinoma cell lines *in vitro*. Following miR-106b transfection, OVCAR3, HO8910-PM, SKOV3/DDP cell lines exhibited (A) significantly slower growth, (B) higher induced G_1_ or S arrest and (C) higher apoptosis than mock-transfected cells. Results are representative of three separate experiments; data are expressed as the mean ± standard deviation, * *P* < 0.05.

**Fig 3:**
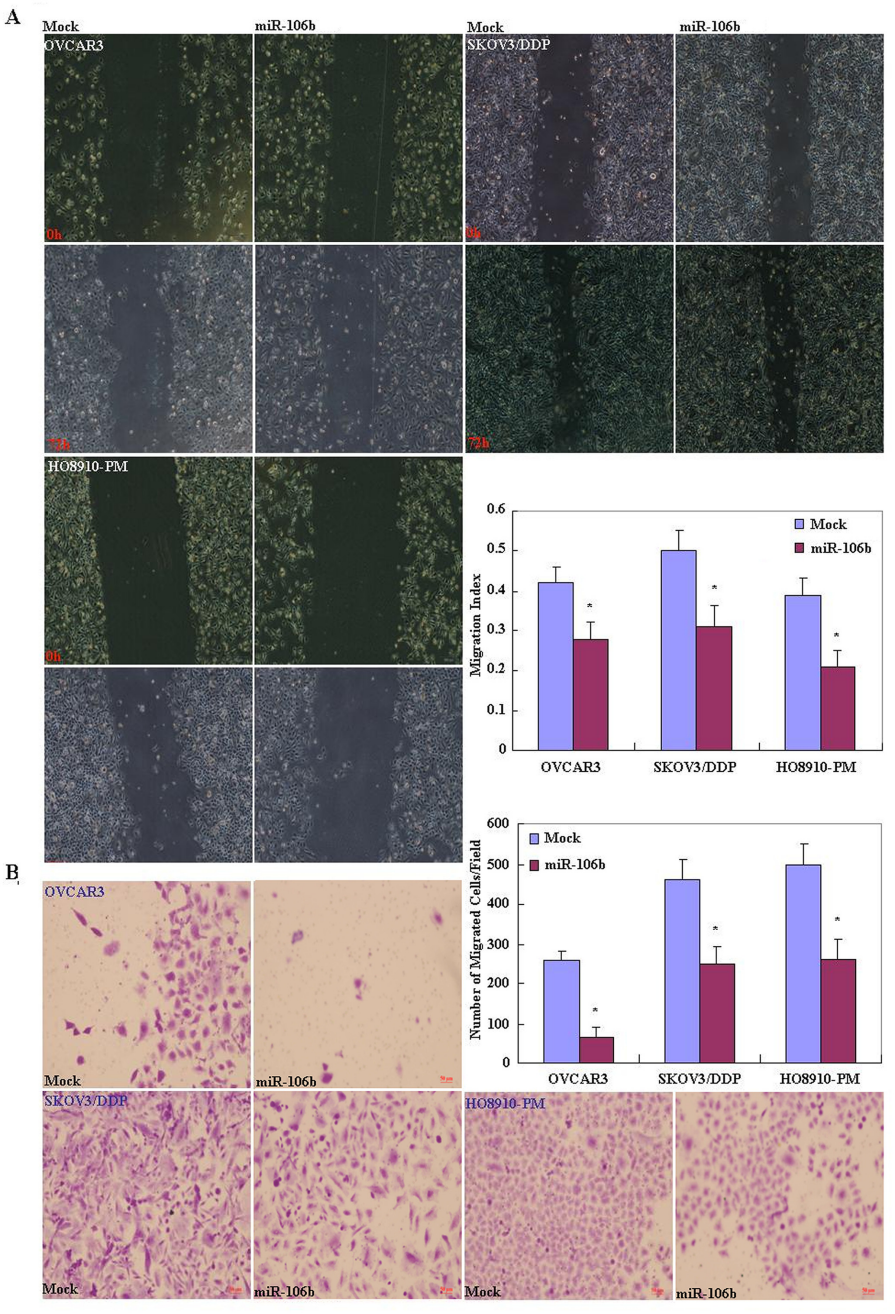
Effects of miR-106b transfection on the invasive and metastatic ability of ovarian carcinoma cell lines. After transfection with the miR-106b mimic, ovarian carcinoma cell lines showed (A) lower migration in wound healing assays, and (B) slower invasion in matrigel transwell assays compared with mock-treated cells. Results are representative of three separate experiments; data are expressed as the mean ± standard deviation, * *P* < 0.05.

OVCAR3, HO8910-PM and SKOV3/DDP cell lines were used for miR-106b transfection. After transfection, the cells exhibited significantly slower growth than mock-transfected (i.e. control) cells (Fig 2A, *p* < 0.05), based on the CCK-8 assay. Propidium iodide (PI) staining and flow cytometry revealed significant induction of G_1_ or S arrest in miR-106b transfectants (Fig 2B, *p* < 0.05). MiR-106b transfection induced significantly higher levels of apoptosis (Fig 2C, *p* < 0.05), indicated by annexin V—fluorescein isothiocyanate (FITC) staining. Reduced cell migration in the wound healing assay (Fig 3A, *p* < 0.05) and reduced invasion in the transwell invasion assay (Fig 3B, *p* < 0.05) were also observed following miR-106b transfection in comparison with mock-transfected cells.

### Effects of miR-106b transfection on genotype in ovarian carcinoma cells *in vitro*

**Fig 4:**
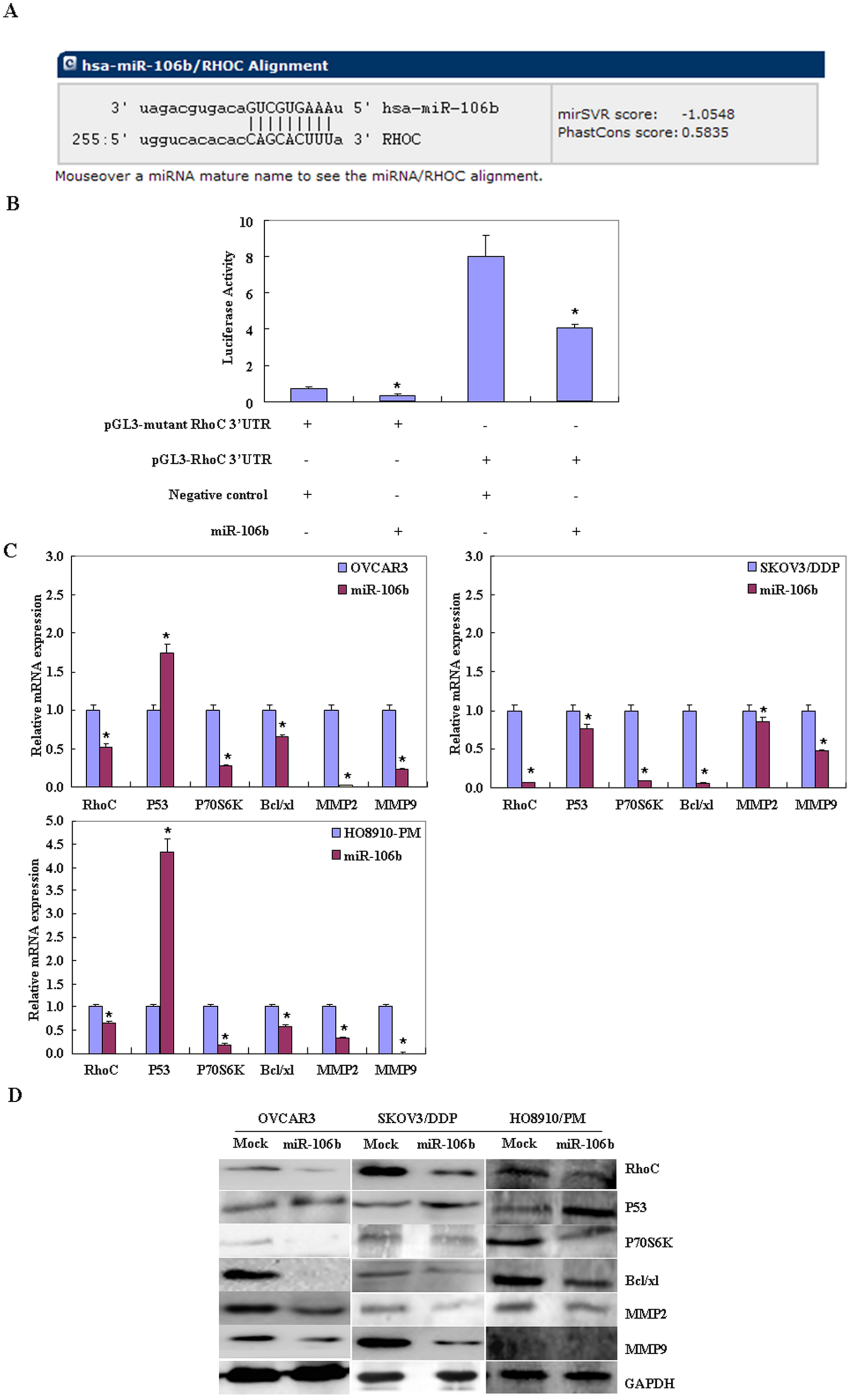
Effects of miR-106b transfection on ovarian carcinoma cell genotype *in vitro*. (A) Analysis of the predicted seed region in the 3’ UTR of *RhoC* showed that *RHOC* was a direct target of miR-106b. (B) The dual-luciferase reporter assay indicated that miR-106b directly targeted *RHOC* by binding to its 3’ UTR. (C and D) MiR-106b overexpression reduced RhoC, P70S6K, Bcl-xL, MMP2 and MMP9 mRNA and protein expression while inducing P53 expression.

The seed region we predicted in the 3’-UTR of RhoC was investigated as a target of miR-106b (Fig 4A). Dual-luciferase reporter assays indicated that miR-106b directly targets RhoC by binding its 3’UTR (Fig 4B). RT-PCR and Western blotting showed that miR-106b transfection reduced mRNA and protein expression of RhoC, P70S6K, Bcl-xL, MMP2 and MMP9, but induced p53 expression (Fig 4C and 4D, *p* < 0.05).

### *In vivo*, miR-106b inhibits tumor growth

**Fig 5:**
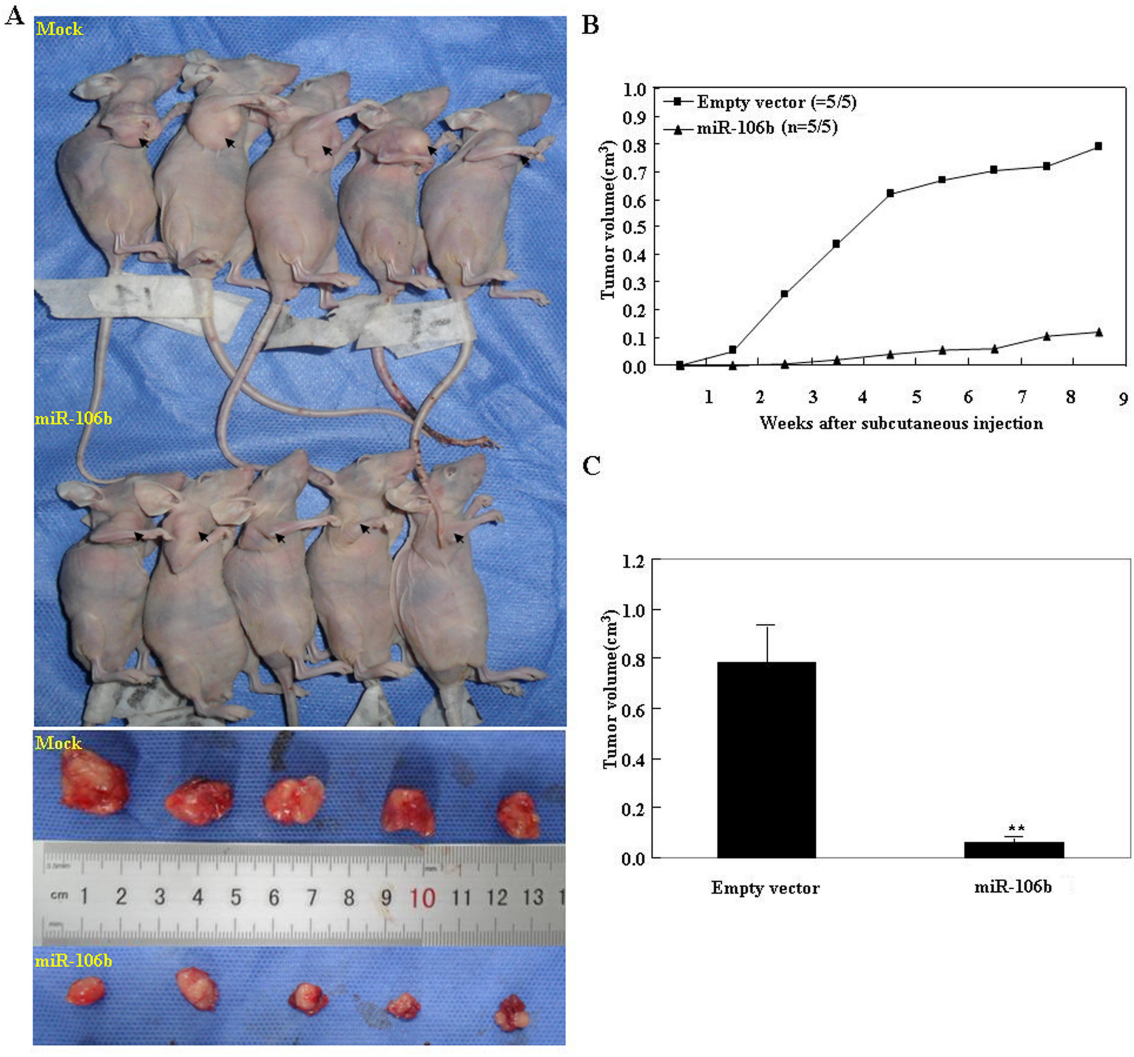
MiR-106b inhibited tumor growth *in vivo*. (A) Tumor xenograft volume in nude mice treated with miR-106b was smaller than in control nude mice. (B) Tumor xenograft growth in miR-106b-treated nude mice was slower than that in the control group from day 7, and (C) the DV increased from week 2 onwards.

The volumes of tumor xenografts in nude mice treated with miR-106b were smaller than those in the control mice (P_day 10_ < 0.05 vs. control; P_deviation of tumor xenograft volume (DV)_ < 0.01 vs. control, Fig 5A). The growth of the tumor xenograft in miR-106b treated mice was slower than in the control group from day 7, and the DV from around 2 weeks (Fig 5B and 5C).

### *In vivo*, miR-106b downregulates RhoC expression in tumor xenografts

**Fig 6:**
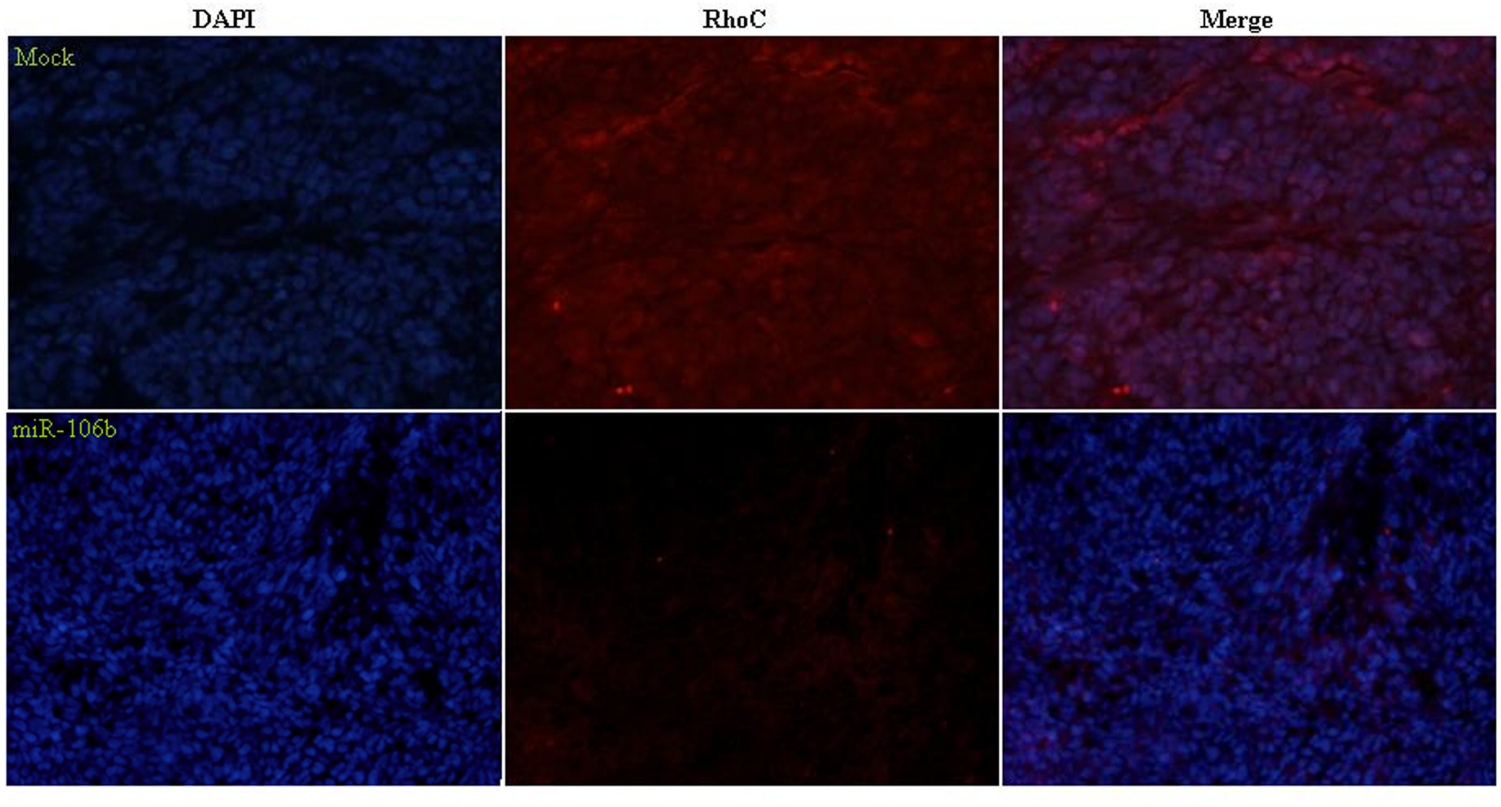
MiR-106b downregulated RhoC expression in tumor xenografts *in vivo*. Immunofluorescent staining indicated that RhoC expression in the tumor xenografts of miR-106b-treated nude mice was decreased compared with that in control (saline-treated) mice.

Immunofluorescent staining analysis indicated that RhoC expression in the tumor xenografts of nude mice treated with miR-106b was decreased compared with that in the tumor xenografts of mice treated with saline (the control group) (Fig 6).

## Discussion

MicroRNAs are promising in methods for cancer treatment and to affect tumor development. MiRNAs contribute to the regulation of their target genes by base-pairing to the 3’UTR of a target mRNA, which results in either mRNA degradation or translation inhibition [9–10]. Deficiency in miRNA expression may contribute to tumor development [11–15]. Studies also show that miRNAs are promising diagnostic and prognostic molecular biomarkers in cancers [16–17]. The results in this study show that miR-106b mRNA expression was significantly lower in ovarian carcinomas and borderline tumors than in benign tumors and normal ovaries. The same result was found in metastatic omentum relative to ovarian carcinomas. MiR-106b mRNA expression was nagatively associated with differentiation (Well *vs*. Por & Mod) and FIGO stage (stage I/II *vs*. stage III/IV) in ovarian carcinoma. These findings indicate that miR-106b might affect ovarian epithelial carcinogenesis and its subsequent progression.

We found that after miR-106 transfection, ovarian cancer cells exhibited significantly slower growth than mock-transfected cells, as well as significantly enhanced G_1_ or S arrest and apoptosis. These data indicate that miR-106 might inhibit the aggression of ovarian carcinoma cells. Additionally, miR-106 transfection decreased P70S6K and Bcl-xL expression, but increased the expression of p53 at both mRNA and protein levels. p70S6K/p85S6K is a downstream effector of the PI3K/Akt/mTOR signal transduction pathway. It phosphorylates the S6 protein of the 40S ribosomal subunit and thus functions in protein synthesis and cell growth. Studies have shown that the p14ARF-MDM2-p53 pathway (p53 pathway) and p70S6K/p85S6K pathway work at the G_1_/S checkpoint, suggesting that miR-106b might reduce cell proliferation and induce G_1_ or S arrest through targeting P70S6K and P53, and induce apoptosis by downregulating the transcription and translation of the anti-apoptotic gene Bcl-xL. MicroRNA 106b-5P overexpression also reduced RhoC, MMP2 and MMP9 mRNA and protein expression. According to the literature, these molecules are involved in the regulation, invasion and metastasis [18–25] of cancer cells.

Ras homolog gene family member C (RhoC) is a small G protein/guanosine triphosphatase which closely linked to tumor growth, metastasis, invasion and progression or adverse prognosis in various malignancies such as head and neck, cervical and gastric cancers [26–30]. Our previous work showed that RhoC could promote the invasion and metastasis of ovarian cancer by affecting VEGF, MMP-9 and ROCK [31]. We also found that after RhoC knockdown, there was reduced mRNA or protein expression of Bcl-xL, survivin and phosphorylated P70S6 kinase (p-p70s6k) [32]. In this work, we show that the 3’-UTR of RhoC is a direct target of miR-106b. In addition, dual-luciferase reporter assays indicated that the relative firefly luciferase activity (normalized with Renilla luciferase) on co-transfection with miR-106b and RhoC was significantly lower than for transfection with RhoC alone, and higher than for miR-106b-transfection-only cells. Taken together, our results suggest that miR-106b may inhibit cell proliferation, induce cell apoptosis, and inhibit cell invasion by modulating the expression of various genes through targeting RhoC.

Furthermore, our tumor xenograft studies *in vivo* showed that miR-106b transfection inhibits tumor growth of ovarian epithelial carcinoma. Immunofluorescent staining analysis indicated that RhoC expression was decreased in tumor xenografts of mice treated with miR-106b compared to tumor xenografts of saline treated mice. These are further indications that miR-106b might suppress tumor proliferation and metastasis by targeting RhoC.

In conclusion, we demonstrate that miR-106b may inhibit ovarian epithelial carcinoma tumorigenesis and progression, by targeting RhoC. The involvement of miR-106b-mediated RhoC downregulation in EOC aggression may provide wider insight into the molecular mechanisms underlying cancer aggression. Approaches aimed at overexpressing miR-106b may serve as promising therapeutic strategies to treat EOC patients.

## Supporting Information

S1 Table The inhibitor role of miR-106b in ovarian epithelial carcinoma. (DOC)

## References

[1] Nam EJ, Yoon H, Kim SW, Kim H, Kim YT, Kim JH, et al MicroRNA Expression Profiles in Serous Ovarian Carcinoma. Clin Cancer Res. 2008; 14(9): 2690–2695. 10.1158/1078-0432.CCR-07-1731 18451233

[2] Pasquinelli AE. MicroRNAs and their targets: recognition, regulation and an emerging reciprocal relationship. Nat Rev Genet. 2012; 13(4): 271–282. 10.1038/nrg3162 22411466

[3] Ambros V. MicroRNA pathways in flies and worms: growth, death, fat, stress, and timing. Cell. 2003; 113: 673–676. 12809598 10.1016/s0092-8674(03)00428-8

[4] Ambs S, Prueitt RL, Yi M, Hudson RS, Howe TM, Petrocca F, et al Genomic profiling of microRNA and messenger RNA reveals deregulated microRNA expression in prostate cancer. Cancer Res. 2008; 68(15): 6162–70. 10.1158/0008-5472.CAN-08-0144 18676839 PMC2597340

[5] Tang Q, Zhong HZ, Xie FY, Xie JY, Chen HM, Yang G. Expression of miR-106b-25 induced by salvianolic acid B inhibits epithelial-to-mesenchymal transition in HK-2 cells. European Journal of Pharmacology. 2014; 741: 97–103. 10.1016/j.ejphar.2014.07.051 25094038

[6] Khuu C, Jevnaker AM, Bryne M, Osmundsen H. An investigation into anti-proliferative effects of microRNAs encoded by the miR-106a-363 cluster on human carcinoma cells and keratinocytes using microarray profiling of miRNA transcriptomes. Front Genet. 2014;5: 246 10.3389/fgene.2014.00246 25202322 PMC4142865

[7] Sampath D, Calin GA, Puduvalli VK, Gopisetty G, Taccioli C, Liu CG, et al Specific activation of microRNA106b enables the p73 apoptotic response in chronic lymphocytic leukemia by targeting the ubiquitin ligase Itch for degradation. Blood. 2009; 113(16):3744–53. 10.1182/blood-2008-09-178707 19096009 PMC2670791

[8] Ni X, Xia T, Zhao Y, Zhou W, Wu N, Liu X, et al Downregulation of miR-106b induced breast cancer cell invasion and motility in association with overexpression of matrix metalloproteinase 2. Cancer Sci. 2014; 105(1): 18–25. 10.1111/cas.12309 24164962 PMC4317878

[9] Bartel DP. MicroRNAs: genomics, biogenesis, mechanism, and function. Cell. 2004; 116: 281–297. 14744438 10.1016/s0092-8674(04)00045-5

[10] Farazi TA, Hoell JI, Morozov P, Tuschl T. MicroRNAs in human cancer. Adv Exp Med Biol. 2013; 774: 1–20. 10.1007/978-94-007-5590-1_1 23377965 PMC3704221

[11] Croce CM. Causes and consequences of microRNA dysregulation in cancer. Nat. Rev. Genet. 2009; 10: 704–714. 10.1038/nrg2634 19763153 PMC3467096

[12] Farazi TA, Spitzer JI, Morozov P, Tuschl T. MiRNAs in human cancer. J. Pathol. 2011; 223: 102–115. 10.1002/path.2806 21125669 PMC3069496

[13] Bueno MJ, Malumbres M. MicroRNAs and the cell cycle. Biochim. Biophys. Acta. 2011; 12: 592–601. 10.1016/j.bbadis.2011.02.002 21315819

[14] Sanchez-Diaz PC, Hsiao TH, Chang JC, Yue D, Tan MC, Chen HI, et al De-regulated MicroRNAs in pediatric cancer stem cells target pathways involved in cell proliferation, cell cycle and development. PloS One. 2013; 8(4): e61622 10.1371/journal.pone.0061622 23613887 PMC3629228

[15] Deng S, Calin GA, Croce CM, Coukos G, Zhang L. Mechanisms of microRNA deregulation in human cancer. Cell Cycle. 2008; 7: 2643–2646. 18719391 10.4161/cc.7.17.6597

[16] Allegra A, Alonci A, Campo S, Penna G, Petrungaro A, Gerace D, et al Circulating microRNAs: New biomarkers in diagnosis, prognosis and treatment of cancer (Review). Int. J. Oncol. 2012; 41(6): 1897–912. 10.3892/ijo.2012.1647 23026890

[17] Sotillo E, Thomas-Tikhonenko A. Shielding the messenger (RNA): microRNA-based anticancer therapies. Pharmacol. Ther. 2011; 131(1): 18–32. 10.1016/j.pharmthera.2011.04.006 21514318 PMC3124007

[18] Stamenkovic I. Extracellular matrix remodelling: the role of matrix metalloproteinases. J Pathol. 2003; 200(4): 448–464. 12845612 10.1002/path.1400

[19] Kessenbrock K, Plaks V, Werb Z. Matrix metalloproteinases: regulators of the tumor microenvironment. Cell. 2010; 141(1): 52–67. 10.1016/j.cell.2010.03.015 20371345 PMC2862057

[20] Gialeli C, Theocharis AD, Karamanos NK. Roles of matrix metalloproteinases in cancer progression and their pharmacological targeting. FEBS Journal. 2011; 278(1): 16–27. 10.1111/j.1742-4658.2010.07919.x 21087457

[21] Moroz A, Delella FK, Lacorte LM, Deffune E, Felisbino SL. Fibronectin induces MMP2 expression in human prostate cancer cells. Biochem Biophys Res Commun. 2013; 430: 1319–1321. 10.1016/j.bbrc.2012.12.031 23261429

[22] Park KS, Kim SJ, Kim KH, Kim JC. Clinical characteristics of TIMP2, MMP2, and MMP9 gene polymorphisms in colorectal cancer. J Gastroenterol Hepatol. 2011; 26: 391–397. 10.1111/j.1440-1746.2010.06504.x 21261731

[23] Lin YL, Ramanujum R, He S. Infection of Schistosomiasis japanicum is likely to enhance proliferation and migration of human breast cancer cells: mechanism of action of differential expression of MMP2 and MMP9. Asian Pac J Trop Biomed. 2011; 1: 23–28. 10.1016/S2221-1691(11)60063-4 23569720 PMC3609145

[24] Yoshizaki T, Sato H, Murono S, Pagano JS, Furukawa M. Matrix metalloproteinase 9 is induced by the Epstein-Barr virus BZLF1 transactivator. Clin Exp Metastasis. 1999; 17(5): 431–436. 10651310 10.1023/a:1006699003525

[25] Ogden A, Rida PC, Aneja R. Heading off with the herd: how cancer cells might maneuver supernumerary centrosomes for directional migration. Cancer Metastasis Rev. 2013; 32(1–2): 269–87. 10.1007/s10555-013-9446-4 23114845 PMC3581755

[26] Liu N, Zhang G, Bi F, Pan Y, Xue Y, Shi Y, et al RhoC is essential for the metastasis of gastric cancer. J Mol Med. 2007; 85(10): 1149–56. 17549441 10.1007/s00109-007-0217-y

[27] Islam M, Lin G, Brenner JC, Pan Q, Merajver SD, Hou Y, et al RhoC expression and head and neck cancer metastasis. Mol Cancer Res. 2009; 7(11): 1771–80. 10.1158/1541-7786.MCR-08-0512 19861405 PMC2887764

[28] Abe H, Kamai T, Tsujii T, Nakamura F, Mashidori T, Mizuno T, et al Possible role of the RhoC/ROCK pathway in progression of clear cell renal cell carcinoma. Biomed Res. 2008; 29(3): 155–61. 18614849 10.2220/biomedres.29.155

[29] Boone B, Van Gele M, Lambert J, Haspeslagh M, Brochez L. The role of RhoC in growth and metastatic capacity of melanoma. J Cutan Pathol. 2009; 36(6): 629–36. 10.1111/j.1600-0560.2008.01117.x 19222696

[30] Kleer CG, Teknos TN, Islam M, Marcus B, Lee JS, Pan Q, et al RhoC GTPase expression as a potential marker of lymph node metastasis in squamous cell carcinomas of the head and neck. Clin Cancer Res. 2006; 12(15): 4485–90. 16899593 10.1158/1078-0432.CCR-06-0376

[31] Zhao Y, Zong ZH, Xu HM. RhoC expression level is correlated with the clinicopathological characteristics of ovarian cancer and the expression levels of ROCK-I, VEGF, and MMP9. Gynecol Oncol. 2010; 116(3): 563–71. 10.1016/j.ygyno.2009.11.015 20022093

[32] Zhao Y, Zheng HC, Chen S, Gou WF, Xiao LJ. The role of RhoC in ovarian epithelial carcinoma: a marker for carcinogenesis, progression, prognosis, and target therapy. Gynecol Oncol. 2013; 130(3): 570–8. 10.1016/j.ygyno.2013.06.004 23764197

