# Supplementary material for: Inhibition of Ovarian Epithelial Carcinoma Tumorigenesis and Progression by microRNA 106b Mediated through the RhoC Pathway
Source: PLoS One. 2015 May 1;10(5):e0125714. doi: 10.1371/journal.pone.0125714 (PMC4416747; doi:10.1371/journal.pone.0125714)
Supplement: S1 Table — (DOC) [file pone.0125714.s001.doc]

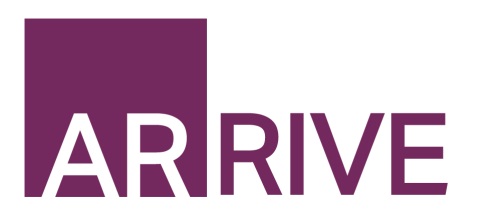


The ARRIVE Guidelines Checklist

Animal Research: Reporting In Vivo Experiments

Carol Kilkenny^1^, William J Browne^2^, Innes C Cuthill^3^, Michael Emerson^4^ and Douglas G Altman^5^

*^1^The National Centre for the Replacement, Refinement and Reduction of Animals in Research, London, UK, ^2^School of Veterinary Science, University of Bristol, Bristol, UK, ^3^School of Biological Sciences, University of Bristol, Bristol, UK, ^4^National Heart and Lung Institute, Imperial College London, UK, ^5^Centre for Statistics in Medicine, University of Oxford, Oxford, UK.*

|  | ITEM | RECOMMENDATION | Section/ Paragraph |
| --- | --- | --- | --- |
| Title | 1 | Provide as accurate and concise a description of the content of the article as possible. | The inhibitor role of mir-106b in ovarian epithelial carcinoma |
| Abstract | 2 | Provide an accurate summary of the background, research objectives, including details of the species or strain of animal used, key methods, principal findings and conclusions of the study. | Ovarian epithelial carcinoma cell lines OVCAR3 (serous cystic adenocarcinoma) with or without miR-106b transfection was injected into the BALB/c female mice axillas subcutaneously to establish xenograft model. This could clarify the function of miR-106b in vivo by measuring and calculating tumor growth velocity and the final volume. As a result, miR-106b has a significantly inhibitory effect on ovarian carcinoma oncogenesis and development. |
| INTRODUCTION | | |  |
| Background | 3 | a. Include sufficient scientific background (including relevant references to previous work) to understand the motivation and context for the study, and explain the experimental approach and rationale.  b. Explain how and why the animal species and model being used can address the scientific objectives and, where appropriate, the study’s relevance to human biology. | Recently researches revealed that miR-106b acted as tumor inhibitor in many kinds of tumors. In vitro study, we have found some interesting results showing the relationship between miR-106b and oncogenesis in ovarian cancer cells. But all these data need to be fully investigated and the miR-106b efficiency against tumor in vivo was proceeded. |
| Objectives | 4 | Clearly describe the primary and any secondary objectives of the study, or specific hypotheses being tested. | The xenografting of miR-106b was carried out to study its effect in ovarian cancer in vivo. Also, the aim of the present study is to explore whether miR-106b underlying an inhibitor role through regulating RhoC. |
| METHODS | | |  |
| Ethical statement | 5 | Indicate the nature of the ethical review permissions, relevant licences (e.g. Animal [Scientific Procedures] Act 1986), and national or institutional guidelines for the care and use of animals, that cover the research. | All animal manipulations were performed strictly in accordance with the National Institutes of Health Guide for the Care and Use of Laboratory Animals, and were approved by the China Medical University Animal Care and Use Committee (Shenyang, Liaoning, China). Mice were sacrificed by CO2 inhalation. All efforts were made to minimum suffering. |
| Study design | 6 | For each experiment, give brief details of the study design including:  a. The number of experimental and control groups.  b. Any steps taken to minimise the effects of subjective bias when allocating animals to treatment (e.g. randomisation procedure) and when assessing results (e.g. if done, describe who was blinded and when).  c. The experimental unit (e.g. a single animal, group or cage of animals).  A time-line diagram or flow chart can be useful to illustrate how complex study designs were carried out. | Ten mice were purchased and assigned into two groups randomly (5 in each group). The xenograft model was established by subcutaneous injection of 200μL PBS containing 5×10^6^ OVCAR3 cells, with or without miR-106b transfection. Tumor volume was measured every three days. |
| Experimental procedures | 7 | For each experiment and each experimental group, including controls, provide precise details of all procedures carried out. For example:  a. How (e.g. drug formulation and dose, site and route of administration, anaesthesia and analgesia used [including monitoring], surgical procedure, method of euthanasia). Provide details of any specialist equipment used, including supplier(s).  b. When (e.g. time of day).  c. Where (e.g. home cage, laboratory, water maze).  d. Why (e.g. rationale for choice of specific anaesthetic, route of administration, drug dose used). | Female BALB/C nude mice (aged 5 weeks, weighing 18 to 20g) were maintained in specific pathogen-free condition (SPF) for rodents. Ten mice were selected and divided equally into two groups randomly. A total of five mice were injected with 5×10^6^ OVCAR3 cells in 0.2ml PBS into the right axillae, while all the other mice were injected with miR-106b transfected cells in amount of 5×10^6^. 7 days after tumor implantation, most of the tumors were palpable (approximately 2*2mm). At week 9 after tumor induction, all the mice were sacrificed by CO2 inhalation. All the tumors were harvested and resected for immunofluroresent staining and western blot. Tumor diameters were measured every three days (length and width in millimeters) through the skin with a caliper and tumor growth curve was calculated according to the formula: TV (tumor volume) (mm3) = length × width2 × 0.5. The final tumor volume was determined by measuring tumor diameters at the end of the experiment. |
| Experimental animals | 8 | a. Provide details of the animals used, including species, strain, sex, developmental stage (e.g. mean or median age plus age range) and weight (e.g. mean or median weight plus weight range).  b. Provide further relevant information such as the source of animals, international strain nomenclature, genetic modification status (e.g. knock-out or transgenic), genotype, health/immune status, drug or test naïve, previous procedures, etc. | For this model, ten female BALB/c nude mice, weighing 18 to 20g, were purchased from Beijing HFK Bioscience Co., Ltd. (China) and maintained in specific pathogen-free circumstance in the Department of Laboratory Animal Science. |

The ARRIVE guidelines. Originally published in *PLoS Biology*, June 2010^1^

| Housing and husbandry | 9 | | Provide details of:  a. Housing (type of facility e.g. specific pathogen free [SPF]; type of cage or housing; bedding material; number of cage companions; tank shape and material etc. for fish).  b. Husbandry conditions (e.g. breeding programme, light/dark cycle, temperature, quality of water etc for fish, type of food, access to food and water, environmental enrichment).  c. Welfare-related assessments and interventions that were carried out prior to, during, or after the experiment. | Mice were kept in accredited facilities under standard conditions for rodents (SPF grade) in the Department of Laboratory Animal Science with adequate sterilized water intake and feed for the animals free. | |
| --- | --- | --- | --- | --- | --- |
| Sample size | 10 | | a. Specify the total number of animals used in each experiment, and the number of animals in each experimental group.  b. Explain how the number of animals was arrived at. Provide details of any sample size calculation used.  c. Indicate the number of independent replications of each experiment, if relevant. | Ten nude mice were selected and randomly divided into two groups (5 per group). | |
| Allocating animals to experimental groups | 11 | | a. Give full details of how animals were allocated to experimental groups, including randomisation or matching if done.  b. Describe the order in which the animals in the different experimental groups were treated and assessed. | Ten nude mice were selected and divided randomly into two groups, which one was injected using normal epithelial ovarian cancer cells and the other group was treated with miR-106b transfected epithelial ovarian cancer cells | |
| Experimental outcomes | 12 | | Clearly define the primary and secondary experimental outcomes assessed (e.g. cell death, molecular markers, behavioural changes). | The final tumor volumes in nude mice treated with miR-106b were smaller than those in the control mice. The growth rate of the tumor xenograft in miR-106b treated mice was slower than in the control group. Immunofluorescent staining analysis indicated that RhoC expression in the tumor xenografts of nude mice treated with miR-106b was decreased compared with the control group. | |
| Statistical methods | 13 | | a. Provide details of the statistical methods used for each analysis.  b. Specify the unit of analysis for each dataset (e.g. single animal, group of animals, single neuron).  c. Describe any methods used to assess whether the data met the assumptions of the statistical approach. | SPSS 10.0 software (SPSS, Chicago, IL, USA) was used to analyze all data. Paired samples t-test was used to compare the means of different groups. p values less than 0.05 were considered to be statistically significant. | |
| RESULTS | | | |  | |
| Baseline data | 14 | | For each experimental group, report relevant characteristics and health status of animals (e.g. weight, microbiological status, and drug or test naïve) prior to treatment or testing. (This information can often be tabulated). | We chose immunodeficient BALB/c nude mice which were generally used in building xenograft models in the world. All the healthy mice came from Beijing HFK Bioscience Co., Ltd. (China) and shared the same sex (female), age (5 weeks), and weight (ranged from 18 to 20g). They have never accepted any treatment or used drug before. | |
| Numbers analysed | 15 | | 1. Report the number of animals in each group included in each analysis. Report absolute numbers (e.g. 10/20, not 50%^2^).   b. If any animals or data were not included in the analysis, explain why. | All the ten nude mice were equally and randomly divided into two groups (5 per group). Due to the appropriate number of cells, the nude mice tolerated the subcutaneous injection well and no animal lethality was observed during the experiment. There was no excluded data or animals in our analysis. | |
| Outcomes and estimation | 16 | | Report the results for each analysis carried out, with a measure of precision (e.g. standard error or confidence interval). | The tumor volumes obtained from miR-106b transfected group were much smaller than those from normal cell group, and significant difference was observed between miR-106b transfected group and normal cell group (p<0.05, Fig. 5A). The growth rate of the tumor xenograft in miR-106b treated mice was slower than the control group (p<0.05, Fig. 5B, C). Immunofluorescent staining analysis indicated that RhoC expression in the miR-106b transfected group was decreased compared with that normal cell group. (Fig. 6). | |
| Adverse events | 17 | | a. Give details of all important adverse events in each experimental group.  b. Describe any modifications to the experimental protocols made to reduce adverse events. | N/A | |
| DISCUSSION | | | |  | |
| Interpretation/ scientific implications | 18 | | a. Interpret the results, taking into account the study objectives and hypotheses, current theory and other relevant studies in the literature.  b. Comment on the study limitations including any potential sources of bias, any limitations of the animal model, and the imprecision associated with the results^2^.  c. Describe any implications of your experimental methods or findings for the replacement, refinement or reduction (the 3Rs) of the use of animals in research. | miR-106b acted as a tumor inhibitor in vivo of epithelial ovarian cancer and can decrease RhoC expression. In this study, we first identified the inhibitor role of miR-106b in epithelial ovarian cancer in vivo and our results revealed RhoC expression level was regulated negatively by miR-106b. | |
| Generalisability/ translation | 19 | | Comment on whether, and how, the findings of this study are likely to translate to other species or systems, including any relevance to human biology. | We first demonstrate that miR-106b may inhibit ovarian epithelial carcinoma tumorigenesis and progression by targeting RhoC via xenograft model of nude mice, which provide wider insight into the molecular mechanisms underlying cancer aggression. | |
| Funding | 20 | List all funding sources (including grant number) and the role of the funder(s) in the study. | | This work was supported by grants from the Liaoning Science and Technology Grant (2013021077) and the National Natural Scientific Foundation of China (81202049, 81472440) |  |


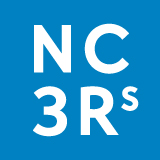


References:

1. Kilkenny C, Browne WJ, Cuthill IC, Emerson M, Altman DG (2010) Improving Bioscience Research Reporting: The ARRIVE Guidelines for Reporting Animal Research. *PLoS Biol* 8(6): e1000412. doi:10.1371/journal.pbio.1000412

2. Schulz KF, Altman DG, Moher D, the CONSORT Group (2010) CONSORT 2010 Statement: updated guidelines for reporting parallel group randomised trials. *BMJ* 340:c332.
